# Citizen Science Based Monitoring of *Greylag goose* (*Anser anser*) in *Bavaria* (*Germany*): Combining Count Data and Bag Data to Estimate Long-Term Trends between 1988/89 and 2010/11

**DOI:** 10.1371/journal.pone.0130159

**Published:** 2015-06-24

**Authors:** Andreas Grauer, Andreas König, Nils Bunnefeld

**Affiliations:** 1 Wildlife Biology and Management working group, Institute of Animal Ecology, Technical University of Munich, Freising, Germany; 2 Biological and Environmental Sciences, University of Stirling, Stirling, United Kingdom; University of Southern California, UNITED STATES

## Abstract

**Introduction, Material and Methods:**

Numbers of large grazing bird (geese, swans, cranes) have increased all over Europe, but monitoring these species, e.g. for management purposes, can be time consuming and costly. In Bavaria, sedentary Greylag geese (*Anser anser*) are monitored during the winter by two different citizen-based monitoring schemes: the International Waterbird Census [IWC] and hunting bag statistics. We compared the results of both schemes for the seasons 1988/89 to 2010/11 by analysing annual indices calculated using the software TRends and Indices for Monitoring Data—TRIM.

**Results and Discussion:**

We identified similar, highly significant rates of increase in both data sets for the entire region of Bavaria (IWC 14% [13–15%], bag 13% [12–14%]). Furthermore, in all of the seven Bavarian regions, trends in annual indices of both data sets correlated significantly. The quality of both datasets as indicators of abundances in Greylag geese populations in Bavaria was not undermined by either weaknesses typically associated with citizen based monitoring or problems generally assumed for IWC and bag data. We also show that bag data are, under the German system of collecting bag statistics, a reliable indicator of species’ distribution, especially for detecting newly colonized areas. Therefore, wildlife managers may want to consider bag data from citizen science led monitoring programmes as evidence supporting the decision making processes. We also discuss requirements for any bag monitoring schemes being established to monitor trends in species’ distribution and abundance.

## Introduction

International agreements oblige member states to maintain naturally occurring wildlife populations, including those of migratory birds, to reduce the loss of biodiversity [1–3]. Because understanding of newly emerging factors regulating distribution and abundance of bird species (e.g., changes in climate and land use) is limited, monitoring is an essential tool to put conservation decisions into action [4,5]. Estimating trends in migratory bird species is complex [6] and most of the existing monitoring schemes have to deal with several problems connected to survey methodology as well as to the ecology, behaviour, and movement of monitored species [5,7]. To acknowledge these problems it is generally recommended to use several independent datasets [8]. Sufficient data from different monitoring schemes is not readily available so that these recommendations can most often not be implemented [9,10].

Monitoring data from two independent datasets, the International Waterbird Census (IWC) [11,12] and hunting bag statistics [13] are available in Bavaria to assess trends in the regional populations of huntable waterbirds. Both statistics measure wintering geese populations; the IWC is carried out during winter and most of the geese are bagged during the months of November, December and January. No analysis of the Bavarian IWC and bag statistics yet exists to test the suitability of the sampling methods for monitoring this mobile and migratory bird species. Data gathered in the Bavarian IWC is heterogeneous because the number of IWC survey sites has increased substantially in the past while some regions are still poorly covered. Furthermore, numbers of surveys per season in the participating survey sites vary due to varying volunteer participation. Short and long term bird migration patterns may also affect trend estimates, e.g., geese may change their staging or wintering area for several reasons [14–16] causing redundant or missing counts.

To use bag statistics as an index of population trends, researchers must acknowledge that hunting bags may not only depend on abundance but also on hunting effort [17–19]. Identified trends may reflect changes in hunting effort rather than trends in species densities. Reliability of hunting bag statistics may also depend on the ability of hunters to identify the species being shot as well as their willingness to report. Nevertheless, for Bavaria there is no evidence that hunters intentionally report either wrong species or wrong numbers. Despite these issues, data quality may also be critical as both monitoring schemes are based on data collected by hunters and bird watchers. Having two independent datasets for one species is an outstanding example of a long-term citizen science based monitoring program, but data should still be scrutinized for their viability [20–22]. With data quality in mind, the aim of the study was to determine whether the trends in the two different Bavarian data sets correlate. If trends in Greylag geese counted and shot do correlate, it would strengthen conclusions based on one of these two datasets.

## Material and Methods

### Greylag geese in Bavaria

Bavaria has never been part of one of Europe’s major flyways or a wintering area for Nordic geese [23]. Local populations were not known until the 1960s [24,25] when Konrad Lorenz and Bavarian hunters tried to introduce the species into the south of Bavaria [24]. Greylag geese breeding populations have spread well since that time. Recent ringing studies state that migration routes are mostly restricted to Bavaria [26,27]. Hence, Bavarian Greylag geese populations must be seen as sedentary populations, though migration patterns of these populations exist [27] and migration routes do connect different regions of Bavaria, an area twice the size as Belgium. We conclude from the recent ringing studies [26,27], that Bavaria’s bag data as well as Bavarian IWC data cover the entire migration route of Bavarian Greylag geese.

### International Waterbird Census

In Bavaria, Greylag geese are surveyed under the framework of the International Waterbird Census (IWC) [28] coordinated by the ornithological subdivision of the *Bavarian Agency for Nature Conservation* (Landesamt für Umwelt; LfU), which is part of the *Bavarian Ministry for Environment* [11]. IWC survey sites cover most of Bavaria, but the *Eastern Low Mountain Range* region is not yet covered at all. Waterbirds are counted by volunteers simultaneously every four weeks during September to April [12]. In this study we used surveys from 1988/89 to 2010/11 and selected data from surveys conducted during November, December and January, i.e., the months that cover the main part of the Greylag geese hunting season in Bavaria. In general, the number of survey sites increased during the time period analysed ranging between 77 and 121 (Table 1). When a survey site had been counted twice during a weekend we used the maximum numbers counted during this time. To obtain one data point per season, monthly results were averaged per survey site using the geometric mean [29]. The numbers of surveys conducted at a specific survey site per season did, however, vary due to changing volunteer participation.

**Table 1:** Numbers of IWC survey sites monitored between 1988/89–2010/11 and number of administrative districts (AD) providing bag data during the same time span.

Region	1		2		3		4		5		6		7		8	
Data Set	IWC	AD	IWC	AD	IWC	AD	IWC	AD	IWC	AD	IWC	AD	IWC	AD	IWC	AD
1988/89	0	14	11	18	1	6	11	20	21	14	18	12	19	9	81	96
1989/90	0		11		1		11		22		17		20		82	
1990/91	0		11		1		10		22		17		21		82	
1991/92	0		11		1		11		22		16		21		82	
1992/93	0		10		1		11		21		16		23		82	
1993/94	0		10		1		12		21		15		19		78	
1994/95	0		10		1		12		21		13		22		79	
1995/96	0		10		1		12		20		13		21		77	
1996/97	0		10		1		12		18		18		23		82	
1997/98	0		10		1		13		20		18		22		84	
1998/99	0		10		1		12		20		18		21		82	
1999/00	0		10		1		13		20		24		23		91	
2000/01	0		11		1		13		21		39		23		108	
2001/02	0		11		1		13		22		41		22		110	
2002/03	0		11		1		11		25		44		22		114	
2003/04	0		11		1		14		25		46		22		119	
2004/05	0		11		1		12		26		49		22		121	
2005/06	0		11		1		11		23		50		22		118	
2006/07	0		10		1		16		21		46		23		117	
2007/08	0		11		1		15		17		47		23		114	
2008/09	0		11		1		15		15		45		24		111	
2009/10	0		11		1		16		14		52		23		117	
2010/11	0		9		1		10		17		48		20		105	

1 = North Eastern Low Mountain Ranges, 2 = Main; 3 = Eastern Low Mountain Ranges, 4 = Upper Danube / Altmuehl, 5 = Lower Danube / Isar, 6 = Southwest Bavaria, 7 = Inn / Salzach, 8 = Bavaria

### Hunting regulations for geese and bag statistics

Under German hunting law the entire territory of Germany is split up into hunting districts [13] and local hunters are, by law, obliged to report the numbers of game bagged or found dead in these hunting districts in paper form shortly after the end of hunting season, latest until April 10^th^ of the following year. Unlike other European states [30] it is a mandatory system for monitoring bag sizes.

The data are submitted separately for each species to the responsible administrations (Bavaria: 96 Administrative Districts, (AD)) every year. The ADs sum the hunting bags of the hunting districts and send the sum to the *Bavarian Ministry of Food*, *Agriculture and Forestry*. The ministry publishes the total bag reached across Bavaria, but stakeholders, like universities or nature conservation related non-governmental organisations, can also obtain detailed statistics [31].

Hunting Greylag geese in Bavaria is not regulated by quotas, so hunters can shoot as many geese as they like to or at least as many as they are able to. Hunting methods are regulated, however, such that birds are only allowed to be shot with rifles of any calibre or shotguns, but they are not allowed to be caught in traps or using nets [3,13,32]. Furthermore, hunters are only permitted to harvest geese during August and between November 1^st^ to January 15^th^, but derogations are possible. The majority of geese are hunted during November and January. Equivalent to the IWC data we used the Greylag goose hunting bags recorded in the ADs during the seasons 1988/89 to 2010/11.

### Geographical reference

**Fig 1:**
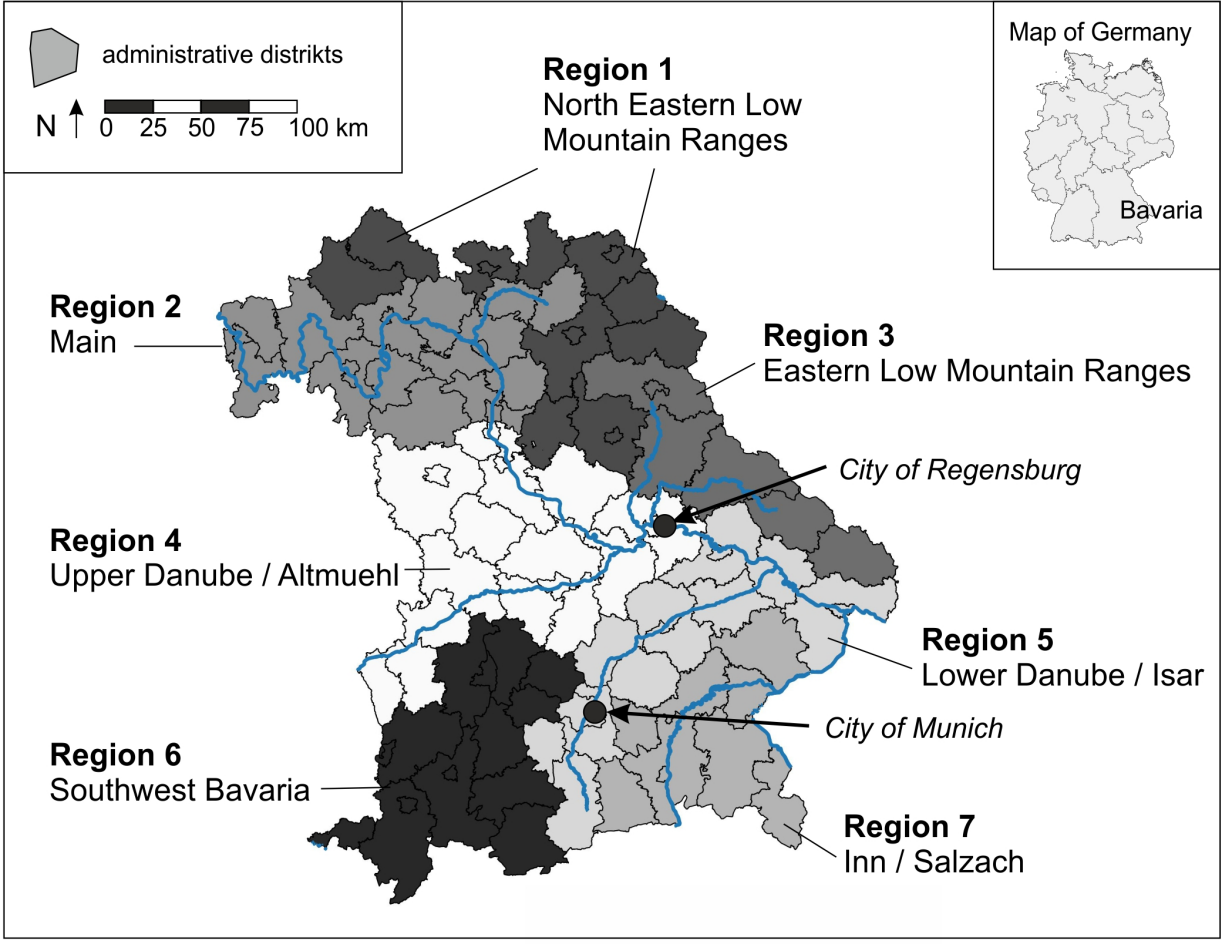
Map of Bavaria including the different regions.

We subdivided the territory of Bavaria (70,550 km²) into seven regions (Fig 1) and assigned IWC survey sites as well as the ADs to those regions (Table 1). All analyses were done regionally and for Bavaria as a whole.

The seven regions were defined according to major rivers systems and landscape structure [33]. The region *North Eastern Low Mountain Ranges* includes ADs in the *Saxony and Thuringia Low Mountain Ranges (Thüringische und Sächsische Mittelgebirge)* as well as the *East Hessian Hills (Osthessisches Hügelland)*. The region *Eastern Low Mountain Ranges* includes the ADs dominated by the low mountain rage “*Bavarian Forest” (Bayerischer Wald)*. Both regions are characterized by forests and some smaller rivers (e.g., *Naab*, *Regen*). The region *Main* includes ADs directly influenced by the course of the *River Main* or the northern parts of the *Altmuehl—Main—Danube—Canal* in which some gravel pits are relevant waterbird habitats in addition to the river course. The region *Upper Danube / Altmuehl* includes all ADs west of the AD *Regensburg* having direct contact to the course of the river *Danube*, the southern parts of the *Altmuehl—Main—Danube—Canal* or the *River Altmuehl*. This region includes major waterbird areas such as the *Lake Altmuehlsee* and large gravel pits along the river *Danube*. The same applies for the region *Lower Danube / Isar* including the courses of the river *Danube* east of AD *Regensburg*, the river *Isar* and some lakes in and south of *Munich* (e.g. *Starnberger See*, *Ammer See*). The city itself also provides many suitable habitats for waterbirds, being the main wintering area for the breeding population of *Lake Altmuehl* [27]. The region *Inn / Salzach* is dominated by the rivers *Inn* and *Salzach* and some lakes like the *Lake Chiemsee*. The region *South-western Bavaria* is strongly influenced by the rivers *Lech* and *Iller*. *Lake Kontanz (Bodensee)* is also situated in the southwest of this region. To a certain extent, the configuration of the regions reflects also the historical occurrence of geese in Bavaria, in that the southern regions cover the area where geese were recorded first and later spread along the river systems [24].

### Analyzing trends in geographical occurrence

**Fig 2:**
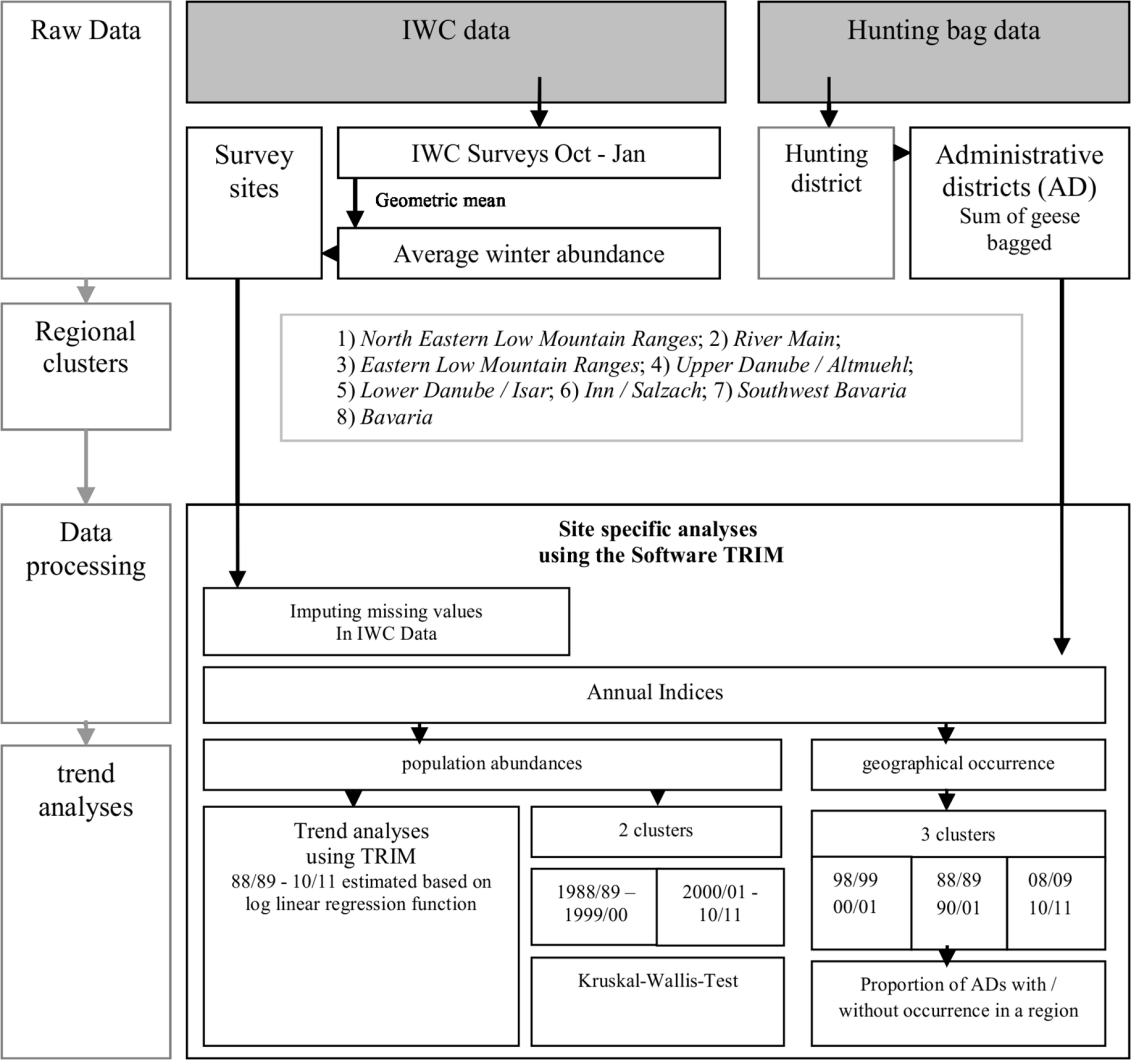
Flowchart of the data processing and analyses (IWC: International Waterbird Census, TRIM: Software

We evaluated trends in the occurrence of Greylag geese in three different 3-year periods, 1988/89–1990/91, 1998/99–2000/01 and 2008/09–2010/11 (Fig 2, Table 2). The chosen 3-year periods are long enough to minimize random variation in the detectability of geese, which would otherwise influence both bag sizes and the IWC data especially in areas where the species does not occur regularly and abundances are low. Furthermore, three years is short enough not to be influenced by any longer-term trends in the data.

**Table 2:** Occurrence of geese in the IWC survey sites and numbers of Administrative Districts, where geese were shot on a regional level and Bavaria in total, seasons 1988/89–1990/91, 1998/99–2000/01 and 2008/09–2010/11.

	data set		IWC Sites	Geese counted			no reports		AD	Geese shot			no geese shot	
Phase	Region		[N]	[n]	[%]	avg. GG/site	[n]	[%]	[N]	[n]	[%]	Avg bag /AD	[n]	[%]
	1	*North Eastern Low Mountain Ranges*	0	-	-	-	-	-	14	1	7.1	0.02	13	92.9
1988/89	2	*Main*	11	0	0	0	11	100.0	18	7	38.9	0.22	11	61.1
-	3	*Eastern Low Mountain Ranges*	1	0	0	0	1	100.0	6	3	50.0	3.89	3	50.0
1990/91	4	*Upper Danube / Altmuehl*	11	0	0	0	11	100.0	20	12	60.0	1.78	8	40.0
	5	*Lower Danube / Isar*	22	1	4.5	0.32	21	95.5	14	11	78.6	3.98	3	21.4
	6	*Southwest* Bavaria	18	3	16.7	4.26	15	83.3	15	9	60.0	5.22	6	40.0
	7	*Inn / Salzach*	22	3	13.6	0.04	19	86.4	9	6	66.7	4.00	3	33.3
		Bavaria	85	7	8.2	1.00	78	91.8	96	49	51.0	2.43	47	49.0
	1	*North Eastern Low Mountain Ranges*	0	-	-	-	-	-	14	2	14.3	0.05	0	0.0
1998/99	2	*Main*	11	6	54.5	5.48	5	45.5	18	10	55.6	1.48	8	44.4
-	3	*Eastern Low Mountain Ranges*	1	0	0	0	1	100.0	6	5	83.3	1.17	1	16.7
2000/01	4	*Upper Danube / Altmuehl*	13	8	61.5	20.50	5	38.5	20	15	75.0	19.82	5	25.0
	5	*Lower Danube / Isar*	21	10	47.6	44.82	11	52.4	14	13	92.9	16.17	1	7.1
	6	*Southwest* Bavaria	39	11	28.2	19.56	28	71.8	15	9	60.0	15.58	6	40.0
	7	*Inn / Salzach*	23	9	39.1	15.58	14	60.9	9	5	55.6	7.74	4	44.4
		Bavaria	108	44	40.7	22.46	64	59.3	96	59	61.5	10.00	37	38.5
	1	*North Eastern Low Mountain Ranges*	0	-	-	-	-	-	14	3	21.4	0.21	11	78.6
2008/09	2	*Main*	11	6	54.5	11.27	5	45.5	18	15	83.3	8.78	0	0.0
-	3	*Eastern Low Mountain Ranges*	1	1	100.0	163.89	0	0.0	6	6	100.0	6.94	0	0.0
2010/11	4	*Upper Danube / Altmuehl*	16	13	81.3	163.89	3	18.8	20	18	90.0	128.25	2	10.0
	5	*Lower Danube / Isar*	18	13	72.2	100.04	5	27.8	14	13	92.9	54.1	1	7.1
	6	*Southwest* Bavaria	56	40	71.4	46.96	16	28.6	15	10	66.7	23.69	5	33.3
	7	*Inn / Salzach*	25	11	44.0	49.71	14	56.0	9	7	77.8	41.00	2	22.2
		Bavaria	127	84	66.1	66.97	43	33.9	96	72	75.0	44.26	24	25.0

IWC = International Waterbird Cencus, AD = Administrative Districts, sites = survey sites providing geese counts, N = Total Number, n = numbers reported, avg. GG/site = average number of Greylag geese reported per site

### Analyzing annual indices and trend analyses

Trends in both IWC and bag data were analysed based on the annual indices calculated by the software *TRends and Indices for Monitoring Data—TRIM*. The software is designed to evaluate monitoring schemes characterised by missing observations [34,35]. The season 1999/2000 was set as the reference.

We also clustered annual indices for bag data and IWC data into two periods, 1988/89–1990/2000 and 2000/01–2010/11 and tested these two groups for significant differences by using the Kruskal-Wallis test (Fig 2).

## Results

### Occurrence / geographical distribution

During the period 1988/89–1990/91 Greylag geese were shot in 51% of the Bavarian ADs covering all of the seven regions in Bavaria. Regional differences exist in the average bag sizes and the number of AD where geese were shot (Table 2). In general, the percentage of ADs that reported bags were quite similar in the regions *Eastern Low Mountain Ranges*, *Upper Danube* / *Altmuehl* and *Southwest Bavaria* (50%–60%), slightly higher in the regions *Lower Danube / Isar* (79%) and *Inn / Salzach* (67%) and substantially lower in the two northern regions, *Main* (39%) and *North Eastern Low Mountain Ranges* (7%). Seasonal bag sizes per AD are relatively small and apart from the region *Southwest Bavaria* (5 bagged geese per AD and season) were never higher than 4 geese/AD.

The IWC data show a diverse picture of geese occurrence in Bavaria in that only 8% of all Bavarian survey sites reported geese. Moreover, IWC data confirm geese occurrence only for the regions *Lower Danube / Isar* (0.32 geese/site), *Southwest Bavaria* (4.26 geese/site) and *Inn / Salzach* (0.04 geese/site). The differences in the regional occurrence identified by the bag statistics are supported by the IWC surveys. The percentage of survey sites reporting geese as well as the numbers of counted geese were highest in *Southwest* Bavaria and lowest in the regions *Lower Danube / Isar* and *Inn / Salzach* reporting (Table 2).

During the following 10 years, geographical occurrence increased in general: 62% of all Bavarian AD reported bags (+ 11%) and 41% of the survey sites (+33%) counted geese during phase 2, 1998/99–2000/01. Despite the general trend, greater increases can be shown for two *Danube* regions. The numbers of IWC survey sites reporting geese in these regions increased by 61.5% (*Upper Danube / Altmuehl*) and 42.1% (*Lower Danube / Isar*). Both regions also had substantial increases in the numbers of AD where geese were bagged (15.0%, 14.3%). Both data sets identify the two *Danube* regions and *Southwest* Bavaria as the centre of Bavarian geese populations during phase 2, 1998/99–2000/01.

The geographic expansion of sites occupied by geese continued during the entire time span analysed here. Hence, in phase 3 (2008/09–2010/11) 66.1% of all Bavarian IWC survey sites reported geese occurrence (+25%) and Greylag geese were bagged in 75% of all AD (+13%). The differences between the regions became larger. In the *Main region* as well as in the region *North Eastern Low Mountain Ranges* bag sizes and number of counted geese increased only to a small extend whereas sightings as well as the number of geese bagged increased more rapidly in the region *Upper Danube / Altmuehl*. The region developed into the centre of the Bavarian goose population, even though geese occurrence increased in other regions (*Lower Danube / Isar*, *Inn / Salzach*, *Southwest Bavaria*, *Eastern Low Mountain Ranges*) as well.

### Trends in IWC

#### Periods

In Bavaria, the IWC survey results recorded during the time span 2000/01–2010/11 were 400% higher than those ones recorded during 1988/89–1999/00 (Kruskal-Wallis test, p<0.001). The regions differed in the scale of the increase (Table 3) and ranged between 178% and 4029%. The differences between the average IWC results of the period 1988/89-1999/00 and the average IWC results during the period 2000/01-2010/11 were highly significant in most of the regions (Table 3).

**Table 3:** Trends in IWC and bag data for Greylag geese in Bavaria, 1988/89 to 2010/11 analysed by TRIM.

	Data	TRIM						difference in periodical averages								
								1988/89-1999/00			2000/01–2010/11			1988/89-1999/00 vs 2000/01–2010/11		
Region		Slope	95% CI		Trend	p	sign	Mean	95% CI		Mean	95% CI		Δ%	P	Sig
		[%]	ll	ul				[Index]	Ll	ul	[Index]	ll	ul			
1. *North Eastern Low*	IWC	No surveys accomplished														
*Mountain Ranges*	Bag	1.33	-9.80	12.46	uncertain			0.48	0.19	0.86	1.45	0.68	2.79	203.56	<0.05	*
2. *River Main*	IWC	10.20	7.24	13.16	strong increase	<0.01	**	0.39	0.25	0.88	1.21	0.92	3.02	212.19	<0.001	**
	bag	6.37	-127.49	140.24	uncertain			1.72	0.20	2.43	2.70	1.63	5.91	57.36	<0.05	*
3. *Eastern Low*	IWC	23.12	6.45	39.79	strong increase	<0.01	**	1.02	0.82	2.64	42.31	10.78	63.44	4029.54	<0.001	**
*Mountain Ranges*	bag	6.57	5,14	8,00	uncertain			1.72	0.24	3.24	2.70	1.49	3.92	57.40	<0.001	**
4. *Upper Danube / Altmuehl*	IWC	20.79	16.26	25.32	strong increase	<0.01	**	0.38	0.23	0.83	4.63	3.26	11.02	1129.78	<0.001	**
	bag	20.03	17.40	22.66	strong increase	<0.01	**	0.42	0.24	0.89	3.23	2.07	7.29	670.39	<0.001	**
5. *Lower Danube / Isar*	IWC	15.21	11.17	19.25	strong increase	<0.01	**	0.35	0.19	0.72	1.56	1.27	4.04	345.76	<0.001	**
	bag	7.34	5.67	9.01	strong increase	<0.01	**	0.63	0.45	1.51	1.22	1.08	3.34	94.73	<0.001	**
6. *Southwest* Bavaria	IWC	10.53	8.41	12.65	strong increase	<0.01	**	0.61	0.45	1.49	1.69	1.24	4.13	178.52	<0.001	**
	Bag	7.62	5,84	10.94	strong increase	<0.01	**	0.63	0.02	1.24	1.22	0,74	1,69	93.65	<0.001	**
7. Rive*rs Inn /Salzach*	IWC	13.93	11.60	16.26	strong increase	<0.01	*	0.67	0.49	1.62	3.21	2.44	8.00	382.74	<0.001	**
	Bag	11.80	8.41	15.19	strong increase	<0.01	**	0.72	0.50	1.69	2.30	1.48	5.19	220.44	<0.001	**
Bavaria	IWC	14.45	13.10	15.82	strong increase	<0.01	**	0.44	0.29	1.01	2.19	1.76	5.63	400.02	<0.001	**
	Bag	13.80	12.78	14.82	strong increase	<0.01	**	0.50	0.34	1.17	2.22	1.52	5.20	341.39	<0.001	**

AD = Administrative Districts, sites = survey sites providing geese counts, 95% CI = 95% Confidence interval, ll = lower level, ul = upper level, Δ% = proportional difference between the average indices, p = p-value for Kruskal-Wallis Test testing difference between the periods

sig * = significant

sig ** = highly significant

#### TRIM

Analysing the IWC data by using the software TRIM showed significant positive increases in the numbers of geese counted for the whole of Bavaria as well as for all of the regions (Table 3). At the state level, an annual increase of 14% (13–15%) was identified in the number of geese counted. Considering the 95% confidence intervals, the average figures of the regions *Eastern Low Mountain Ranges* (23%), *Lower Danube / Isar* (15%) and *Inn / Salzach* (13%) did not deviate significantly from the Bavarian trend, whereas the annual increase in the *Upper Danube / Altmuehl* region (20%) was significantly higher.

A significantly smaller increase was analysed in the region *Southwest Bavaria* being 10% (8–12%). In the *Main region* the regional 95% confidence interval (7–13%) overlaps with the 95% confidence interval of the Bavarian trend (13–15%) and thus significant differences between both increase rates cannot be assumed. The overlapping sector is, however, very small at about 0.06%.

### Trends in bag statistics

#### Periods

Hunting bags increased in Bavaria highly significantly by 341% (Kruskal-Wallis test, p<0.001) in the time span analysed in this study, as the bag index rose from 0.5 (0.3–1.1) in 1988/89–1999/00 to 2.2 (1.5–5.2) in 2000/01–2010/11. The increases in the regions’ bag statistics were also significant and ranged between 57% and 670%. In the Region 1 (203%) and also in Region 2 (57%) increase rates in bag statistics had not been highly but still significant (Table 3).

#### TRIM

The seasonal bag size in Bavaria was 257 Greylag geese in the hunting season 1988/89 and 4843 geese in the hunting season 2010/11. TRIM calculated a highly significant linear increase of 13% (12–14%) within the data set. Whereas in Region 1 (1%), Region 2 (6%) as well as in Region 3 (6%) significant trends are not identified by TRIM, calculated trends for Region 4 (20%), Region 5 (7%), Region 6 (7%) and Region 7 (11%) show a highly significant increase in bag numbers.

### Comparison of IWC and bag indices

**Fig 3:**
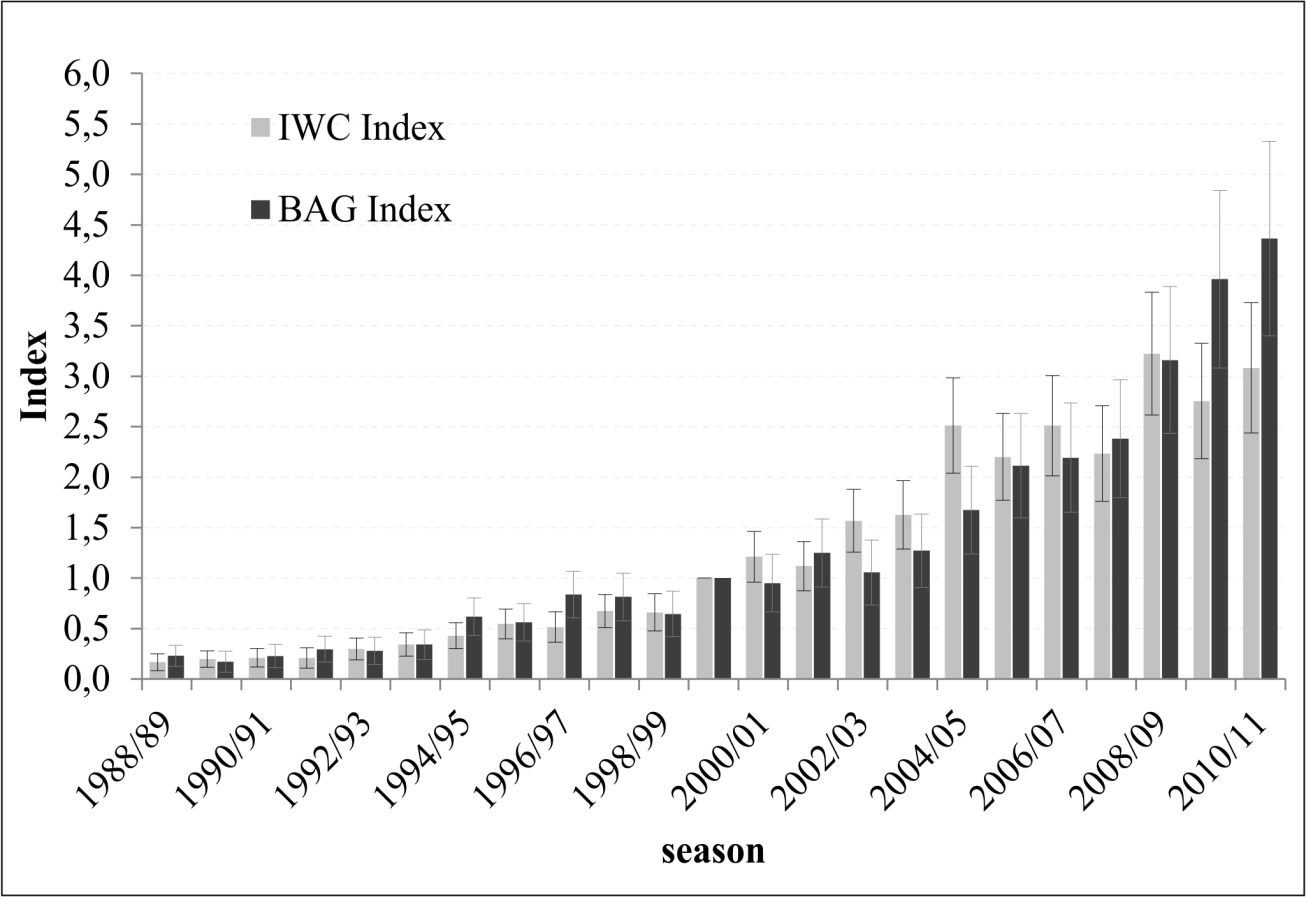
Trends in the annual indices of both, the IWC data and the hunting bag data.

Annual indices in bag and IWC data provided by TRIM show a highly significant positive correlation (Fig 3, Spearman's rank correlation coefficient, R^2^ = 0.97, p<0.001). TRIM calculated almost identical trends for both IWC and bag statistics with increase rates of 14% (13–15%) in the IWC data and 13% (12–14%) in the bag statistics. Furthermore, the 95% confidence intervals of both figures are with ±9% (IWC) and ±7% (bag) very narrow. The similar trend in both statistics is also supported by average indices of the periods 1988/89-1999/00 and 2000/01–2010/11 (Table 3). Comparable to the overall situation, trends in IWC and bag indices of the regions 4, 6 and 7 correspond well. The rates of average annual increase in both datasets are similar proven by relatively narrow and overlapping confidence intervals (Table 3). Furthermore, the average indices of the two periods do support the assumption of almost similar trends in both data sets for those regions. In the region *Main* as well as in the region *Eastern Low Mountain Ranges* confidence intervals of the calculated IWC trends do still overlap, but mostly because one of the confidence intervals, either in the IWC or in the bag data, is considerably large. Looking at the average indices of the two periods indicated no significant differences between the two data sets for the *Main* region, thus it must be assumed, that there is no significant difference between the trends in IWC and bag data here. In contrast, comparing these average indices suggests that the increase in the number of geese bagged is smaller than the increase in the number of geese counted in the region *Eastern Low Mountain Ranges*.

In Region 5, annual trends in both datasets calculated by TRIM do significantly differ as there is no overlap in the 95% confidence intervals. But, as the 95% confidence intervals for the average indices of the periods 1988/89-1999/00 and 2000/01-2010/11 do overlap, it is still questionable if trends in the IWC and bag data do significantly deviate.

Even if the 95% confidence intervals of the average figures overlap, the correlation coefficients for the dependency of IWC and bag statistics are highly significant (Spearman's rank correlation coefficient, p<0.001) with an R^2^> 0.84 in most regions. Only in Region 2 the correlation of IWC and bag indices (R^2^ = 0.41) is not strong, but with p = 0.025 still significant (Table 4). Thus, whenever there is an increase in the IWC counts there is always an increase in the bag statistics, or vice versa. Furthermore, even if there are mostly no significant differences in the growth rates of IWC and bag statistics it seems to be a general tendency that increase rates in bag data seems to be just a little smaller than in the IWC data.

**Table 4:** Spearman's rank correlation coefficient of annual indices in bag and IWC data (N = 23).

Region	R^2^	P
2 *Main*	0.41	p = 0.025
3 *Eastern Low Mountain Ranges*	0.84	p<0.001
4 *Upper Danube* / *Altmuehl*	0.97	p<0.001
5 *Lower Danube / Isar*	0.86	p<0.001
6 *Southwest* Bavaria	0.89	p<0.001
7 *Inn / Salzach*	0.92	p<0.001
Bavaria	0.97	p<0.001

## Discussion

### Trends in Bavaria's Greylag geese population

The value of citizen science for ecological research is now widely accepted [20,21,36], but studies investigating the usefulness and quality of long-term data sets already collected by citizen science schemes for scientific monitoring are rare [37]. Here, we analyse two sets of long-term data collected by two different groups of citizens, the International Waterbird Census (IWC) count data and bag data collected by hunters, spanning the period 1988/89–2010/11 with a total of 23 years of data collection. Besides obvious advantages of gathering data from large areas it is also generally assumed that the collected data may have weaknesses connected to methodological bias. Detectability, unrepresentative distribution of survey sites, survey effort and hunting intensity and/or efficiency as well as qualification of volunteers are known to be able to undermine the quality of monitoring schemes [4,6,17,38–41]. Thus, methods must be established to assure monitoring is effective and minimises bias and error [42]. For the IWC dataset, this is ensured by the organising bodies in Bavaria [12,43] and bag data are collected by qualified hunters receiving training including an examination before they are allowed to hunt [13,32]. Still, identified trends may still be the result of other factors such as changes in the wintering and staging sites of geese and areas not surveyed [5,15]. One of the crucial points influencing monitoring of waterfowl is the relocation of bigger flocks as a result of disturbance, e.g., by hunters [15,44,45]. For Bavaria it has been demonstrated that Greylag geese leave wintering sites from time to time for unknown reasons [27], sometimes geese leave the area of the administrative district, but mostly they do not shift to another region. Thus, it can be assumed that trend analyses carried out at a regional level for bag statistics are not susceptible to these relocations because geese shot in the neighbouring AD will be reported as well. On the other hand, IWC analyses are more liable to be influenced by these relocations because the chances are small that geese will shift to another area that is also being monitored.

Despite these challenges in monitoring, IWC and bag data trends in Bavaria strongly correlate. TRIM indices of bag data and IWC data showed almost identical trends in Bavaria with an annual rate of increase around 13–14% p.a. from 1988/89 to 2010/11 doubling numbers every 5 years. The growth rates we identified are common for (re-)establishing populations of Greylag geese in the last 30 years in other areas. For example, growth rates of about 10% are known for *Great Britain* [46,47] and growth rates of 19% were calculated for Greylag geese populations in *The Netherlands* [48].

As both statistics were independently collected and correspond well, it can be assumed that overall trends in Bavaria are most likely driven by changes in population size rather than by monitoring error or bias. The influence of changes in hunting pressure as well as the influence of changes in hunters’ numbers on trends are often discussed [8,10]. For Bavaria changes in hunting pressure as a result of increasing numbers of hunters may have modified bag numbers during the time analysed in that study. Though official data on numbers of hunters are not available, the total sum of fees which hunters have to pay for hunting related projects when enquiring their licence (20 Euros per person per year) is available and may serve as an indicator of the number of hunters in Bavaria. The total amount of those fees increased slightly from just under 1.1 Million Euros in 2003 to about 1.2 million Euros in 2013 [49] reflecting a slightly positive trend in hunters.

Due to the good fit of IWC and bag data it seems that these methodological and data problems, as well as challenges due to the behaviour of Greylag geese, are successfully addressed for Bavaria. Also at the regional level these challenges have mostly been addressed by the quality of the data. Highly significant correlations of regional indices as well as corresponding rates of increase were identified in the regions *Upper Danube / Altmuehl*, *Southwest Bavaria* and Rivers *Inn / Salzach*. Nonetheless, there are also two regions with slightly poorer correlations of IWC and bag data. For example, in the region *Lower Danube / Isar* the growth rate in the hunting bag statistic is significantly smaller than the one in the survey results. This can be explained by the fact that hunting is forbidden in the city of Munich which is the main wintering area for the quickly increasing population of *Lake Altmuehlsee* consisting of 80 reproducing pairs of *Greylag geese* and about 1000 non-breeding geese in the year 2010 [27]. Hence, most of the population remains unhunted as most of these birds migrate shortly after chicks fledge to Munich just before the hunting season opens [27]. Hence, the discrepancy is connected to legal issues and not to systematic biases. Still, TRIM states a significant strong increase in both data sets for this region and annual indices correlate significantly over the entire study period. Another region contradicting the overall trend is the course of the *River Main* in the north of Bavaria. The reason for the poor fit of growth rates in the indices is the small size of the regional population: on average less than 9 geese are bagged per annum in each administrative district and less than 12 geese are counted per survey site. So all in all, we assume that IWC and bag statistics are equivalent tools to monitor trends in Greylag geese populations in Bavaria.

The analysis of the spatial distribution data over the 23-year period demonstrates the strength of bag data in that these were able to detect newly established goose sites earlier than the IWC in areas where hunting is not restricted. For example, analyses of the bag data and IWC data showed different patterns in the beginning of the time analysed (1988/89-1990/91). Bag data identified a 7-times larger distribution than IWC data and we believe that bag data is the more reliable data set for the following reasons. First, when a goose population is established, the probability of detecting this small number of individuals is, in general, very small. This is especially the case when fixed surveyed periods are set, like the IWC weekends [50,51]. Harvesting birds from these small populations is neither limited to a short period of time by the German hunting regulations nor are any bag limits set. Thus, geese could be hunted in every hunting district covering the entire state of Bavaria. While the IWC is carried out only on major waterbird habitats, hunters collect a continuous harvest record and thus record of species occurrence.

We have shown that both data sets identified similar core areas of geese occurrence. According to both schemes *Southwest Bavaria* (Region 6) and *Inn / Salzach* area (Region 7) were the centre of Bavarian Greylag goose populations during 1988/89–1990/91. When geese populations grew continuously during the next two decades (1988/89–2010/11) differences in the two statistics got smaller, indicating that the earlier discrepancies were the result of small geese abundances causing problems in detectability. It is worth mentioning that both statistics indicate a shift in the core areas of geese occurrence from *Southwest Bavaria* (Region 6) and *Inn / Salzach* area (Region 7) in 1988/89–1990/91 to the two *Danube* regions (regions 4 and 5) in (2008/09–2010/11). Although the reason for the shifts are unknown they may be due to an increasing number of gravel pits left unexploited and being naturalised and thus providing suitable habitats all along the river *Danube*.

### Adjusted management of sedentary Greylag geese populations: Hints from this case study

Parallel to the increasing Arctic populations, populations of Greylag geese have established all over Europe and their numbers are estimated to have increased to about 410,000 Greylag geese in Northwest Europe and to about 31,000 Greylag geese in Central Europe from 1990 to 2010 [36]. Even though most parts of Europe are connected to the different flyways of Nordic Greylag geese populations, some areas still have more or less sedentary regional Greylag geese populations, e.g., the south of England or the south east of Germany, Italy, and Spain [23,52]. Sustainable use of waterfowl populations has long been an issue in wildlife ecology and management [53,54], though there is still substantial uncertainty about system dynamics and impacts of potential management decisions [55]. The two main problems are the almost total lack of reliable data on recruitment and mortality [56]. Therefore, a practical approach is to identify the sustainability of hunting waterbirds by combining bag sizes and population trends [57]. Still, this simple approach is most often restricted by the absence of knowledge about trends in abundances as well as the lack of knowledge of areas of occurrence. This case study has demonstrated that in the case of missing IWC data, bag statistics may be a good indicator for trends in abundances. As European sedentary populations of Greylag goose are mostly hunted and bag data is to some extent available [58,59], these may serve as an indicator for trends within the sedentary populations.

In those regions where bag data are not available [58,59], a system to collect bag data should be established in the future. Elmberg et al. [56] as well as Mooij [59] advocate for long term, standardized schemes to monitor harvest size. As a result of our practical knowledge such monitoring schemes need to fulfil several preconditions to provide data that can be used to identify trends in populations: (a) the bag statistics need to cover the entire region of a regional population, and (b) bag statistics need to have a clear geographical reference. With respect to data quality, it is recommended that hunting can take place everywhere, not being restricted to specific areas. Furthermore, it needs to be considered that bag data is more precise the bigger the population sizes are because larger populations reduce the impact of detection (shooting) probability. When population sizes, e.g., in *Main region*, are very small, trends in bag sizes seem to underestimate population trends. Thus, besides being a reliable indicator of area of occurrence and trends, bag data are also useful as an indicator of population abundances when direct counts are missing. On the other hand, if IWC data is available and hunting is banned, but considered to be allowed as part of an adjusted wildlife management this case study demonstrated that decision-making bodies can rely on IWC data to identify the main areas of occurrence. This knowledge provides the opportunity to set up diverse hunting regulations for areas of different wintering abundances. For example, in core areas of occurrence, hunting may not be limited by numbers, but regulated by areas where geese numbers are low. Nonetheless, our case study suggests that hunting may not always be able to modify trends in populations of sedentary *Greylag geese*.

## Supporting Information

S1 File Bag data per administrative district per season. (TXT)

S2 File IWC data per survey site per season. (TXT)
